# Modification of Lightweight Aggregate Concretes with Silica Nanoparticles—A Review

**DOI:** 10.3390/ma14154242

**Published:** 2021-07-29

**Authors:** Karol Federowicz, Mateusz Techman, Myroslav Sanytsky, Pawel Sikora

**Affiliations:** 1 Faculty of Civil and Environmental Engineering, West Pomeranian University of Technology in Szczecin, al. Piastów 50a, 70-311 Szczecin, Poland; mtechman@zut.edu.pl (M.T.); pawel.sikora@zut.edu.pl (P.S.); 2 Department of Building Production, Lviv Polytechnic National University, S. Bandera Str. 12, 79013 Lviv, Ukraine; myroslav.a.sanytskyi@lpnu.ua; 3 Building Materials and Construction Chemistry, Technische Universität Berlin, Gustav-Meyer-Allee 25, 13355 Berlin, Germany

**Keywords:** lightweight concrete, strength, durability, nanosilica, nanoparticles

## Abstract

The use of lightweight concrete (LWC) for structural and non-structural applications has attracted great interest in recent years. The main benefits include reduced deadload of structural elements and generally lower production and transportation costs. However, a decrease in concrete density often leads to a decrease in strength and durability. Typically, concretes are mostly modified with mineral additives such as silica fume or fly ash. Because of the recent developments in nanotechnology, research attention has turned to the possibility of improving concrete properties with nanomaterials, i.e., nano-SiO_2_. However, there are still certain issues with the dosage and efficiency of nanomaterials. Therefore, in order to establish the current state of knowledge in this field, this review gathers most recent results about the performance of LWC modified with nanomaterials. The review is divided into sections about the influence of nanoparticles on the fresh properties of concrete and their influence on the mechanical and durability characteristics. The paper studies in depth the most common approach to nanomaterials in concrete technology and proposes areas for further development.

## 1. Introduction

### 1.1. Lighweight Concrete—General Consideration

**Figure 1:**
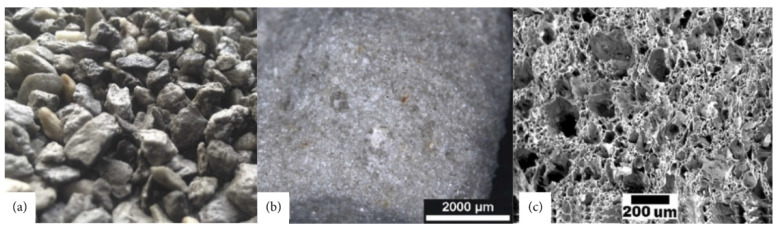
Natural LWA perlite: (**a**) macro scale, (**b**) micrograph to 32×, and (**c**) SEM micrograph to 500×, adapted with permission from ref. [7].

**Figure 2:**
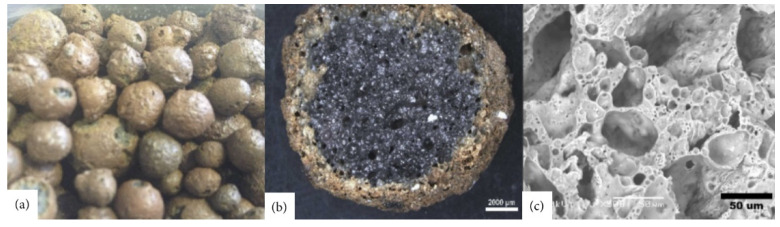
Artificial LWA produced from expanded clay (aliven): (**a**) real scale, (**b**) micrograph to 32×, and (**c**) SEM micrograph to 500×, adapted with permission from ref. [7].

Waste management is a crucial factor for decreasing environmental contamination. One of the best solutions to minimize this issue is reusing and recycling the materials and by-products from various branches of the industry. A good example of recycling is the production of fly ash (FA), where 19 M tons in 2012 were produced in just one European country—Turkey (3% of world’s total). It is projected that the amount of FA production could grow by more than 30% by 2020 [1,2]. One of the many solutions to prevent the depletion of natural resources and decrease environmental pollution is the production of artificial aggregates. This helps to dispose industrial waste and decrease the usage of natural aggregates in the building industry [3,4]. Among the many available types of artificial aggregates, the most popular are lightweight aggregates (LWA) such as Poraver (Germany), Leca (Denmark), or Liapor (Germany). The above mentioned aggregates differ in terms of raw materials used for their production, water absorption, and strength, but have one thing in common: a low density due to the high porosity, as shown in Figure 1 and Figure 2. The concept of lightweight concrete (LWC) can be dated back to 3000 years ago [5]. The Mediterranean region is filled with structures constructed with LWC, including the famous Pantheon Dome built in the early Roman Empire [6]. Historical LWC was made with natural lightweight aggregates such as pumice, diatomite, or scoria, with grains of variable shape and quality. The availability of these aggregates limited the spread of LWC to the Mediterranean Sea basin [5].

Lightweight concrete (LWC), according to the EN 206-1 [8], is defined as having a density below 2000 kg/m^3^. LWCs can be divided into six density classes, from 800 kg/m^3^ with a grading of 200 kg upwards [9]. The use of lightweight aggregates for the production of concrete has many advantages [10,11,12]. Some of these can particularly include a reduced dead load, high thermal efficiency, and enhanced resistance against fire [13]. There are many methods of producing lightweight concrete (LWC), one of which is based on omitting the fine portion of the total concrete aggregate. Another approach is to use chemical admixtures to produce stable air bubbles in the cement matrix and mechanical foaming. This type of concrete is known as aerated, cellular, or gas concrete. The most common method of LWC production is by using lightweight aggregates [14].

Lightweight aggregate concrete (LWAC), in addition to the above-mentioned advantages, exhibits a lower early-age shrinkage compared with ordinary concrete, caused by the internal curing effect [15,16], lower transportation costs of precast units [17], and reduction in formwork and propping [18]. Typically, materials such as expanded shale, clay, or slate materials are used as lightweight aggregates for structural applications as a result of their porous structure obtained through firing [19]. As mentioned before, the application of lightweight aggregate has many benefits to the environment, which are important for the current generation. LWAs and their production help in the management of some industrial waste and in reducing the usage of natural aggregates, limited in natural deposits. Limited exploitation of natural resources saves riverbeds from activities and reduces CO_2_ emissions. It also means a lower specific energy consumption due to the limited use of cement, one of the major carbon dioxide producers [20,21].

In previous decades, lightweight aggregate concrete (LWAC) has been successfully developed and improved. The main interest has been focused on lightweight cementitious composites with a density lower than 1500 kg/m^3^ and sufficient mechanical strength for structural applications [19,22,23]. Even though the potential application of LWAC in structural areas seems promising, it is important to maintain the balance between properties such as the density, strength, stiffness, and durability. This is the greatest challenge that hinders the wide spread application of LWAC in construction engineering. The main disadvantage of LWAC is the lower compressive strength, higher deformability, and weaker bonding strength between the cement matrix and the LWA compared with ordinary concrete [24,25,26]. Lightweight aggregates have a lower strength and elastic modulus than the mortar paste, and are therefore also lower than normal-weight concrete [25]. Another problem that strikes researchers is the fact that no systematic mix design methodology for LWC has been addressed, especially considering a balance between the mechanical properties and thermal properties [27].

In order to address the above-mentioned issues and increase the durability of LWAC, researchers have looked towards mineral additives. LWAC can be easily modified by supplementary cementitious materials (SCMs), which can improve the microstructure and thus the overall strength and durability [22]. The concrete industry is putting a heavy focus on sustainability, for example, by producing low-cement concrete through partially replacing it with SCMs. This approach increases the economic and ecological value, as cement production requires high amounts of energy [28,29] and great amounts of CO_2_ are emitted during its production process. About 90% of the cumulative energy needed for concrete production is spent during the production of cement [28,30]. In the past, many scholars have investigated the role of different SCM in concrete. Industrial waste and by-products such as silica fume (SF), fly ash (FA), and slag (S) are commonly used in concrete [31,32,33]. However, silica fume is one of the most popular pozzolans [34] at a micro-scale. It has been proven that silica fume is one of the most effective SCMs for improving the properties of LWAC [35,36,37]. As a pozzolanic material, silica fume reacts with the Ca(OH)_2_ and produces additional calcium silicate hydrate (C-S-H) gel. Additional C-S-H gel results in a denser microstructure, which leads to an improvement in the properties of hardened cementitious materials [38,39].

### 1.2. Recent Research Progress and Fields of Interest

Optimization of the mixture designs of LWCs towards the production of high-performance materials with improved mechanical properties, as well as resistance against aggressive environments, is the main topic of interest for researchers and industry. Lightweight high-performance concrete (LWHPC) elements have a distinctive fine pore structure and overall low porosity that corresponds to an improvement in the mechanical properties and durability [40,41]. The properties of LWHPC are strongly correlated to the structure of the paste and paste−aggregate interface, which are commonly modified by incorporating admixtures and additives [42,43].

The permeability of concrete is considered as a representative property related to the overall durability of concrete [44,45]. Because the majority of concrete elements are reinforced with rebars, some innovative studies have recently been conducted to determine the structural behaviours of steel reinforced LWHPC elements [46,47,48,49,50]. Another approach proposed by various authors focuses on the production of fibre-reinforced lightweight concrete [51,52,53,54,55]. The abovementioned studies have succeeded in obtaining lightweight concrete with a low density and thermal conductivity, while retaining the high mechanical properties. A lack of natural coarse aggregate typically leads to a lower elastic modulus, lower stiffness, and could result in increased shrinkage and creep. As mentioned before, the permeability of LWC, which indicates the durability of a material, is correlated to the amount, shape, and connection between the pores, as well as the quality of the paste. Nyame [56] found that cement composites made with lightweight sand have an almost doubled permeability compared with those made with natural sand, and this could lead to significant reduction it general durability. Al-Khaiat et al. [57] stated that the chloride ion concentration that penetrated into the LWC structure was significantly higher than that in a traditional concrete, which also highlights the need for modifying the paste structure.

Lightweight concrete, because of its low density, is also a good insulator; the general scope of the thermal properties of lightweight concrete was summarized by Loudon [58]. He reported that, even though thermal conductivity depends on the density and moisture content, the properties of aggregate can also influence it by up to 25%. Zhang and Gjørv [59] reported that the cement paste can penetrate lightweight aggregates during mixing, but the strength of that effect depends on the microstructure of the aggregate surface, particle size distribution of cement, and paste viscosity. Demirboğa et al. [60] conducted study on the thermal conductivity and compressive strength of lightweight concrete with expanded perlite aggregate modified by mineral additives. The authors found that SF and FA used as a cement replacement decreased the thermal conductivity up to 15%, simultaneously reducing the density and compressive strength of the concrete by up to 30%. That research indicates a very important issue about the LWC: increasing the insulation properties by decreasing the density often leads to a decrease in mechanical properties.

Yu et al. investigated the durability of ultra-lightweight concrete composite (ULWC) mixes with the fine LWA that had a low open porosity [27]. They found that the evaluated ULWC mixes had sufficient resistance to water and chloride ions, even though the total porosities of ULWCs was higher than in ordinary concrete. This effect could be related to the internal closed-pores in LWA particles. This type of pore structure reduces the transportation of water, while the permeability of the material is correlated to the quality of cement paste. Liu et al. [61] developed a LWC with a low water and chloride-ion permeability. The design mix had 500 kg/m^3^ of cement and a density of 1400 kg/m^3^. A low density was obtained thanks to use of the expanded clay and expanded glass. The 28-day compressive strength of the concrete reached 24 MPa, which is sufficient for structural applications.

Ling et al. [62] studied lightweight concrete bricks containing expanded polystyrene (EPS) and rice husk ash (RHA) as lightweight aggregates. The authors found that the replacement of cement by 10 wt.% RHA gave the best results. These were found to be the same as that for traditional concrete water curing, which was suggested to be the most effective curing method. Another interesting research work was done by Akçaözoglu et al. [63]. The researchers studied lightweight concrete with waste PET as LWA. The use of PET pellets not only allowed for utilising industrial waste, but also resulted in a reduction of the thermal conductivity of concrete to 0.4–0.6 W/m/K, while normal-weight concrete typically has thermal conductivity of approximately >1.0 W/m/K. In the study, the authors obtained a density between 1530 and 1930 kg/m^3^ by substituting the NWA with PET by 30% to 60% of its volume. The corresponding compressive strength at 28 days varied between 9.5 to 25.3 MPa. The former mix had the potential to be used as an insulation material, while the latter could be considered for the production of structural elements.

### 1.3. Nanoparticles as Novel Admixtures to LWC

**Figure 3:**
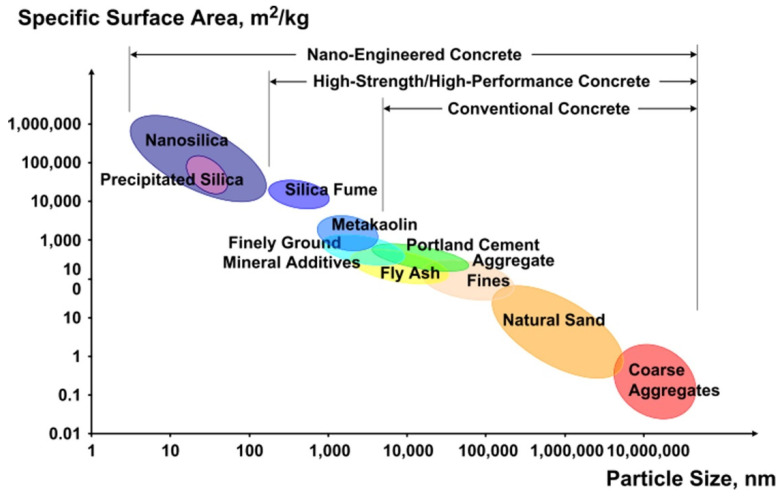
Particle size and specific surface area related to concrete materials, reprinted with permission from ref. [74] Copyright 2010, Elsevier.

**Figure 4:**
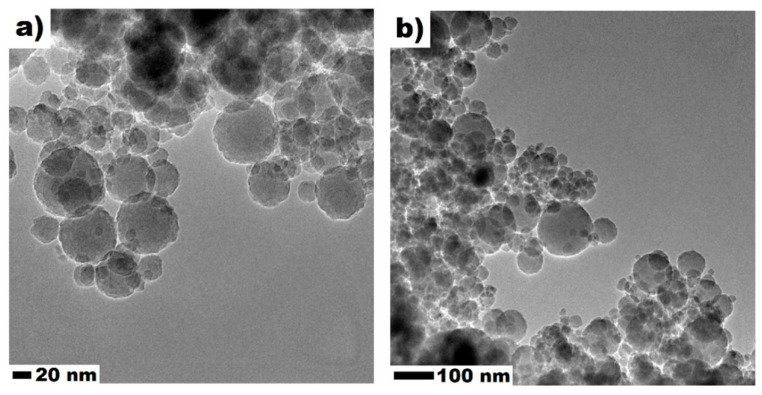
Transmission electron microscope (TEM) micrograph of nanosilica (NS) in high (**a**) and low (**b**) magnification, adapted with permission from ref. [75].

To date, the incorporation of micro-sized SCMs into LWC has been widely researched. In recent years, there has been growing interest in nano-scale particle applications in LWC technology [64,65]. These particles significantly influence the properties of cementitious composites thanks to their fine sizes and chemical and physical properties [66]. Typical nano-scale particles are nano-TiO_2_, nano-Fe_2_O_3_, nano-Al_2_O_3_, nano-SiO_2_, carbon nanotubes/fibers [67], and zinc oxide nanoparticles [68]. Among all these nanoparticles, nanosilica (NS) is the most suitable and commonly used for the production of cementitious materials. The amorphous structure and high production purity (more than 99%), as well as the high specific surface area, are among the biggest advantages (Figure 3) [66,69,70,71]. Exemplary transmission electron microscope micrographs of NS are presented in Figure 4. The TEM images clearly show NS’s spherical shape for particle sizes below 100 nm [72]. Depending on the type of nanoparticles, the dosage can vary from very low, i.e., 0.01 wt.%, up to 10 wt.%. The relatively high cost of nanoparticles limits their industrial-scale applications in construction [73]; therefore, there is a strong need to optimize the nanoparticle content in the mixtures in order to both benefit from their presence and reduce their financial impact on the concrete price.

Nanosilica is known to be an extremely reactive additive [76,77]. It accelerates the hydration of cement by working as the nucleation site for the formation of C-S-H gel. The NS pozzolanic activity increases the total generation of C-S-H in the matrix. Additionally, NS fills the gaps, decreasing the water absorption and allowing for the improvement of the durability of the cementitious matrix [78,79]. Available studies show [80,81,82,83,84] that NS exhibits a significantly higher pozzolanic activity than the silica fume.

Various approaches towards the incorporation of NS in cement-based composites have been proposed. These include either the addition of NS in the form of a dry powder or a colloidal suspension. The colloidal form of NS has a much better dispersity compared with the powder, increasing its overall efficiency [67]. At an early age, the cement hydration rate can be accelerated by additional nucleation sites, which will result in increased early-age strength of cement composites [83,85,86]. The application of NS could reduce the porosity of the cementitious materials by producing calcium-silicate hydrate (C-S-H) gel from Portlandite. This influences the rate of calcium extraction and decreases the accessibility for water and chloride ions [87,88,89]. Quing et al. [90] reported that NS significantly improves the strength and durability, and influences the microstructure of cement paste compared with other pozzolanic materials. Li [91] reported that for fly ash, which normally has a low initial activity, even small amounts of NS notably increased its pozzolanic activity. The reaction of nanoparticles with calcium hydroxide crystals produces an arrayed C-S-H gel in the interfacial transition zone (ITZ) between hardened the cement paste and aggregate. Compelling results were reported by Qing et al. [90]. The authors stated that 3 wt.% NS enhances the strength of the ITZ significantly better compared with the SF. This occurs by consuming the calcium hydroxide, decreasing the orientation of its crystals, and reducing their size at the zone.

Although, the effects of nanosilica on the properties of normal-weight cement-based composites have been widely studied and reviewed by many researchers, studies related to the LWC are still limited. To date, there have been several studies reporting the influence of NS on the properties of LWC, but no guidelines or summaries have been proposed. Therefore, this review aimed to fill the gap in the state-of-art and presents the recent developments in the field of modification of LWC with NS, as well as to propose further directions and possible applications for the concrete industry.

## 2. Fresh Properties of Lightweight Concrete Modified with Nanosilica

To date, many studies related to the rheological properties of cement-based composites modified with nanosilica are available [92,93,94]; however, there are still many uncertainties about the rheological performance of LWC modified with NS. This issue is highly important, as because of the high differences between the density of concrete’s components, LWC exhibits a tendency to segregation and bleeding. The LWC mixtures are often designed to be self-compacting to exclude the need for compaction during execution in order to decrease the risk of segregation. Therefore, particular care has to be taken during the design of LWC mixtures containing NS. Table 1 represents the studies related to the influence of NS on the fresh properties of LWC, as well as the testing methods.

**Figure 5:**
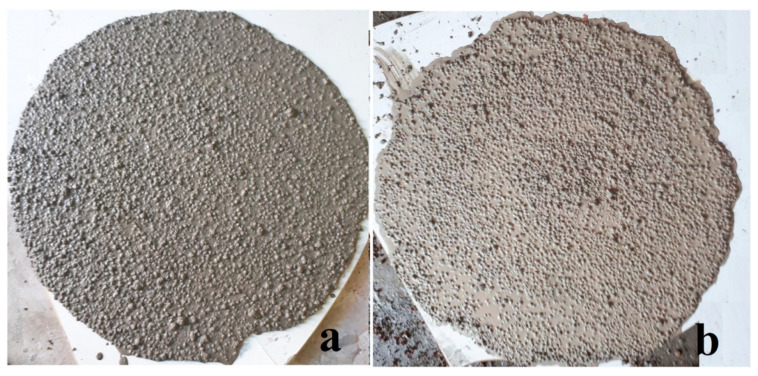
Appearance of the LWCs after the flow test: (**a**) Mix with 3 wt.% NS and (**b**) mix without NS, reprinted with permission from ref. [23] Copyright 2018, Elsevier.

Based on the cited articles, it is clear that the superplasticizer dose needs to be increased with the increase in the NS content. A simultaneous decrease in the stabilizer dosages with the increase in NS content was required in order to achieve a reasonable flowability. This is caused by the fact that the viscosity of the fresh mixture would increase with the increment of NS dosage, as shown in Figure 5. According to Atmaca N. et al. [96], all of the LWCs were designed for a slump flow of 15 ± 2 cm in order to ensure easy mixing and forming. To maintain it, the authors increased the dose of the superplasticizer with the addition of NS. Similar findings were published by Naniz O. et al. [23], where the addition of colloidal nanosilica reduced the slump flow of the prepared mixes. In general, nano-SiO_2_ particles adsorb partially water molecules, negatively influencing the workability. Water molecules are typically attracted to the NS particles because of their high affinity and high specific surface area. This leads to a state where increasing the w/b ratios decreases the required dosage of SP. At the same time, increasing the w/b ratio results in a decrease in density. For instance, the optimum dosage of SP in a mixture with 1 wt.% NS changed from 9.45 kg/m^3^ (density of 1910 kg/m^3^) to 4.05 kg/m^3^ of SP (density of 1878 kg/m^3^), while the w/c ratio changed from 0.35 to 0.45. This is because the available free water increased with the increase in the w/c ratio. On the contrary, adding NS (3 wt.% according to Naniz O. et al.) eliminates bleeding and segregation in LWC. This concurred in the findings of Jalal et al. [97], who state that the incorporation of silica fume and nanosilica influenced the consistency of the concrete mixtures. Another study showed that a small amount of NS could result in an increase in the flowability of concrete [98,99,100,101]. This is due to “ball bearing” effect, where spherical-shaped particles of SiO_2_ can slightly reduce the frictional forces among the particles and improve packing, which leads to an increase in the accessible lubricating water.

The V-funnel test, for which the results were presented by Naniz et al. [23], was used to assess the viscosity and filling ability of the fresh concrete mix. An increase in the amount of NS and SF in the mix increased the viscosity and cohesiveness of the mixes, which led to a decreasing filling ability. This agreed with Güneyisi et al. [80]; they reported that the V-funnel time increased with the increased NS content. In addition, Bernal et al. [102] stated that mixtures showed an increased V-funnel time with the increase of NS content. It is worth noting that increasing the NS contents in the mixes increased the V-funnel time, regardless of the amount of SP. This is because the addition of mineral particles with a high specific surface area enforced the increase of water to keep the workability of the fresh concrete [44].

**Figure 6:**
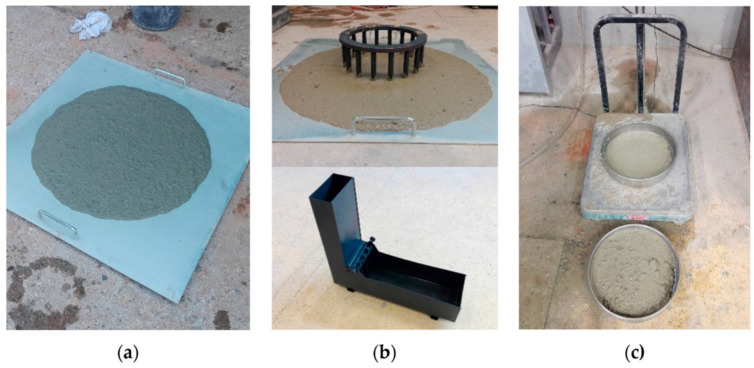
Test of fresh self-compacting concrete: (**a**) flowability and viscosity (slump flow test), (**b**) passing ability (J-ring and L-box), and (**c**) segregation resistance (sieve segregation test), reprinted with permission from ref. [103].

Another method for testing the rheological properties is the J-ring test, presented in Figure 6, where the passing ability of concrete is checked. As seen in Table 1, the results of the J-ring test showed the same correlation as the slump flow test. The use of nanosilica in the concrete mixes decreased the J-ring diameter.

The U-box test is a method used to assess the filling and passing ability of concrete. The test reflects the conditions where the concrete needs to fill a formwork with dense reinforcement. Similar to previously described tests, the majority of mixes containing nanosilica performed worse in the L-Box test. This means that the addition of NS decreased the filling and passing ability. Analysing the results summarized in Table 1, it is worth noting that the effect of NS dosage varied between the mixes because of their different composition, but a general trend can be easily observed.

The use of increased amounts of NS requires a simultaneous increase in the SP content of up to 22 kg/m^3^, which can eventually result in a higher cost of produced concrete. With the increase in SP dosages, the total amount of viscosity modifying admixtures (VMA) should be decreased, as it influences the workability in a similar manner to NS. Therefore, various parameters should be taken in account when designing LWCs. The necessary amounts of SP required for maintaining the rheological properties of fresh mix often exceed the optimal and maximal dosage recommended by the manufacturers. Moreover, a high dosage of polycarboxylate (PCE) plasticizer can result in a retardation of the hydration process, which limits its use in prefabricated concrete. Therefore, calorimetric studies should be considered as a supplementary tool to evaluate the combined effect of NS and PCE on the hydration process and setting time of mixtures.

## 3. Influence of Nanosilica on Mechanical Properties

To date, NS was found to have a spectacular effects on the mechanical performance of NWC in early age, as well as its long-term strength. The nucleating effect of silica nanoparticles [104] results in an earlier formation of the C-S-H phase, influencing the thixotropic properties and early strength development of mortars. The strength of this effect depends on the amount of nanosilica, form (colloidal or powder), and mixture composition. As mentioned before even though there are many articles about the nanosilica effect in normal-weight concrete, the different failure mechanism of lightweight concrete exposes new areas for further investigation. Studies related to the effects of NS on the mechanical performance of LWCs are summarized in Table 2.

**Figure 7:**
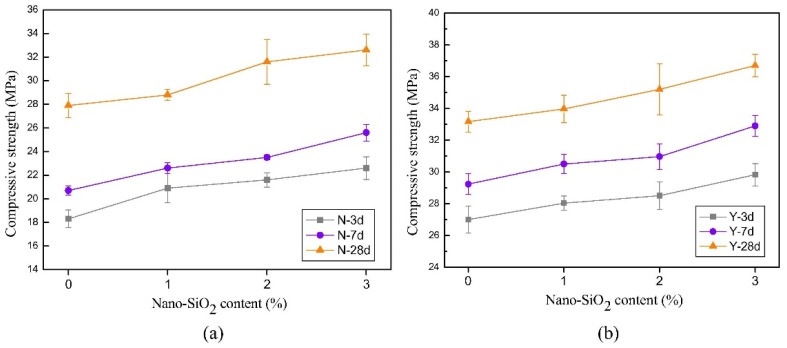
Compressive strength of LWAC with different nano-SiO_2_ dosages: (**a**) fly ash-clay ceramsite and (**b**) shale ceramsite, reprinted with permission from ref. [22] Copyright 2018, Elsevier.

As mentioned above, most research confirms a positive influence of nanosilica on the mechanical properties of concrete. Based on the collected literature data, it was found that the compressive strength of mixes containing NS was higher than the control ones. The effect of the NS content on the strength development of LWC is presented in Figure 7. Several studies have shown that increasing the dosage of NS, usually above 5 wt.%, does not result in any significant improvement in concrete strength. Moreover, it was observed that the strength can decrease due to the particle agglomeration in the matrix [73,100,105]. The increase in the mechanical properties caused by the presence of NS is related to the nuclei that bond with cement hydrate, improving the hydration [72]. It is worth mentioning that this phenomenon can be also reported in blended cementitious systems. For instance, the NS can accelerate the pozzolanic activity of fly ash, resulting in an increased production of C-S-H gel [106]. Moreover, in lightweight concrete, there are unfilled microvoids in mixes due to the typically porous structure of the artificial aggregates. Those voids act as weak spots, lowering the compressive strength in comparison with normal aggregates. Natural, non-porous aggregates are covered completely with cement paste in contrary to porous artificial lightweight aggregates. This leads to a lower bonding strength in the interfacial transition zone around the aggregate. Based on the results cited in Table 2, it is clear that the NS dosage is also related also to the w/b ratio. For the majority of cases, the optimal usage is below 5 wt.%.

According to Naniz et al. [23], replacing the cement by 3 wt.% NS results in an increase of compressive strength by approximately 31.4% and 28.8% for w/b ratios of 0.35 and 0.45, respectively. Other studies on self-compacting concretes and mortars have also reported combining nanosilica and silica fume presents better results than adding only one of the additives [97,107]. It should be noted that in most studies, replacing higher amounts of cement with NS, from 3 wt.% to 5 wt.% (and more), decreased the compressive strength. This effect could be caused by the agglomeration of NS particles, which leads to the formation of weak zones [108].

**Figure 8:**
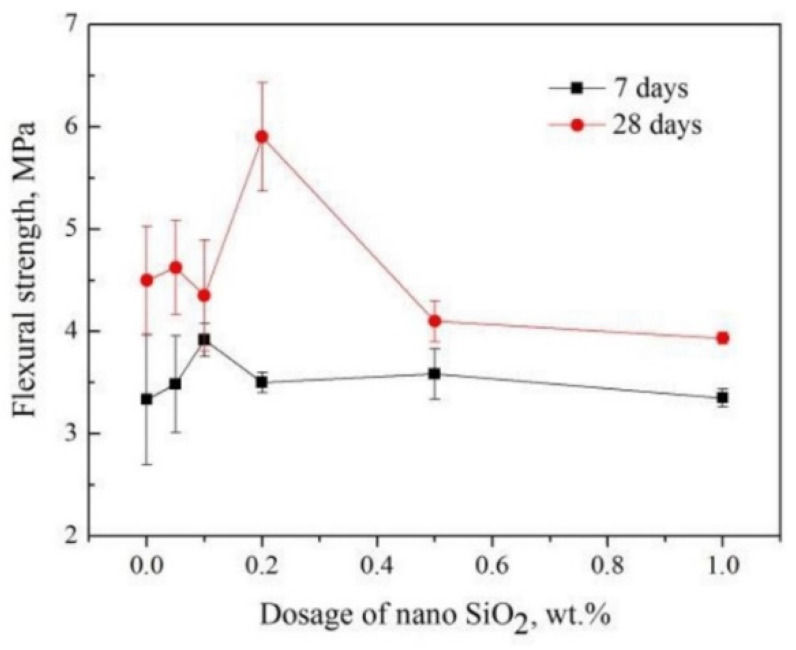
Flexural strength of lightweight concrete containing different low NS dosages after 7 and 28 days of curing. Adapted with permission from ref. [73].

Similar to the compressive strength, the splitting tensile strength and flexural strength of concrete have been improved by the addition of NS (Table 2). The effect occurs regardless of the dosage, age of concrete (7, 28, and 90 days), and amount of lightweight aggregates. According to Abd Elrahman et al. [72], a beneficial effect on flexural strength development was observed for 2 wt.% and 4 wt.% NS, which is coherent with other studies [23,73,96]. The specimens with NS exhibited a higher flexural strength after 7 and 28 days of curing, gradually increasing with the amount of NS added to the mixture. Interesting conclusions were made by Zhang et al. [73]—the peak values of the 7-day compressive strength and flexural strength were achieved for an NS dosage of 0.1–0.2 wt.%. By comparing to the control sample, the 7-day compressive strength and flexural strength increased by 40% and 18%, respectively (Figure 8). These findings indicate that even seemingly insignificant amounts of nanosilica can significantly improve the mechanical properties of concrete.

**Figure 9:**
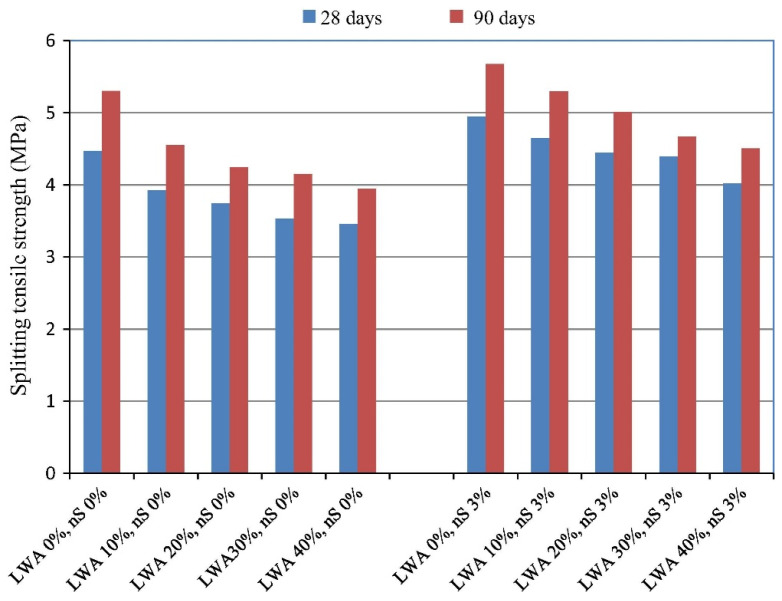
The effect of the LWA and NS content on the splitting tensile strength of high-strength LWC. Reprinted with permission from ref. [96] Copyright 2017, Elsevier.

Similarly, Atmaca et al. [96] reported a substantial influence of NS on the splitting tensile strength of high-performance lightweight concretes, regardless of the LWA content (Figure 9). For instance, after 28 days of curing, LWC containing 40% LWA and 3 wt.% NS achieved over an 16% higher splitting tensile strength than the reference LWC. Similar results were obtained for the modulus of rupture, meaning that is also influenced by the presence of NS [96].

**Figure 10:**
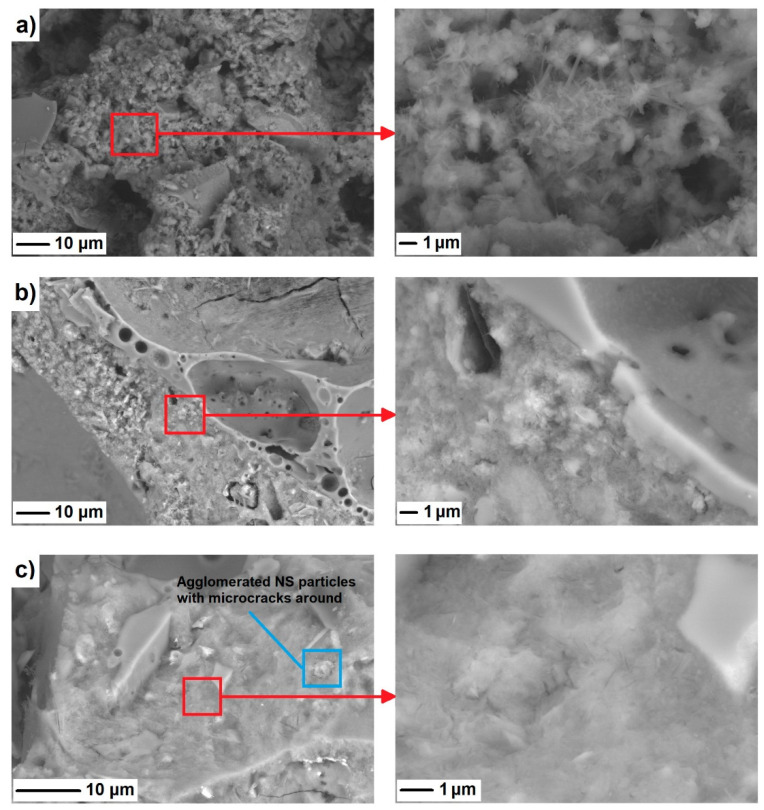
Scanning electron microscope micrographs of a sample (**a**) without NS, (**b**) with 2 wt.% NS, and (**c**) 4 wt.% NS after 28 days of curing, reprinted with permission from ref. [72].

An interesting approach was presented by Vargas et al. [7]. According to their previous work [76], 10 wt.% nanosilica (suspended silica) was observed to be the optimal replacement for cement. They proved that the compressive strength increased considerably. In addition, the pore network decreased and its tortuosity increased as well [76]. In newer research of the same research group [7], the addition of nanosilica did not increase the compressive strength. This could be caused by many reasons, but mainly because LWC exhibited failure through the aggregate not the matrix [7], contrary to what happens in the ordinary concrete. It should also be noted that at higher NS dosages, the finest particles tended to agglomerate, which resulted in microcracking occurring around the agglomerated particles, caused by the volumetric changes associated with drying. As a result of the agglomeration, so-called weak-zones were produced in the concrete, limiting the further increase of its mechanical performance (Figure 10) [72].

**Figure 11:**
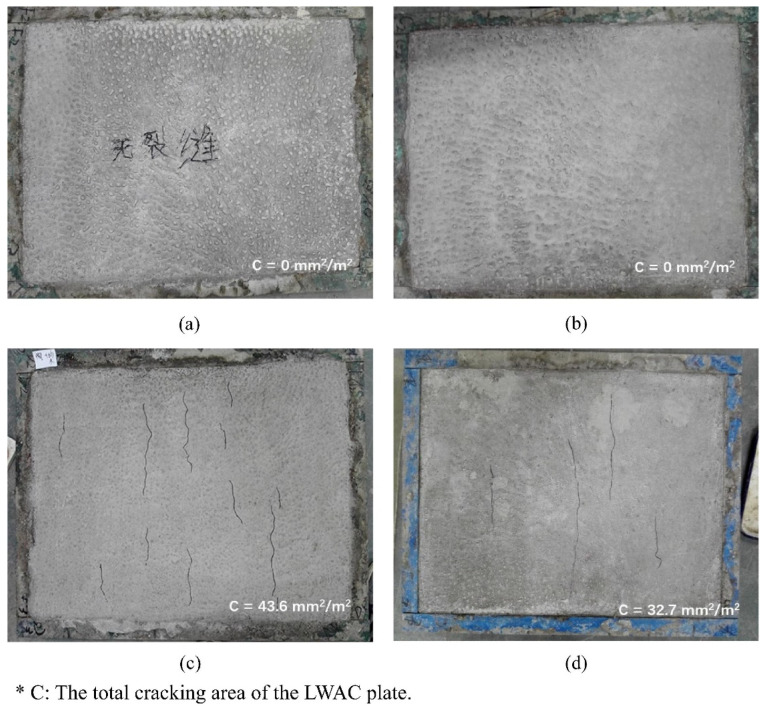
Cracking of LWAC with different nano-SiO_2_ dosages: (**a**) fly ash-clay ceramsite, 0 wt.% nano-SiO_2_; (**b**) fly ash-clay ceramsite, 3 wt.% nano-SiO_2_; (**c**) shale ceramsite, 0 wt.% nano-SiO_2_; and (**d**) shale ceramsite, 3 wt.% NS, (Chinese words in (**a**) is samples notation and it is not essential for this paper) reprinted with permission from ref. [22] Copyright 2018, Elsevier.

Whenever the mechanical properties are in consideration it is worth to mention and discuss the aspect of shrinkage. The effect of lightweight aggregate on total shrinkage is well studied. Because of the internal curing effect, LWA helps to reduce the total shrinkage [109,110]. In terms of shrinkage in lightweight concrete with NS, the amount of research is limited. One of the reasons might be the fact that NS might have a rather negligible effect on the total shrinkage, as proven by Wang et al. [22]. According to their findings, the effect of nano-SiO_2_ on shrinkage was statistically insignificant. During first 7 days, all the shrinkage curves were almost identical. The difference in the value of shrinkage was visible after 90 days, the average measured total shrinkage increased by 2.2%, 3.9%, and 5.4% for LWAC with ceramsite from Nantong with 1 wt.%, 2 wt.%, and 3 wt.% nano-SiO_2_ addition, respectively [22]. The rate of shrinkage development was similar for all of the studied mixes. At this point, it should be emphasized that even if the total shrinkage and its rate were not affected by nanosilica, adding NS particles helps minimise the surface cracking, as shown in Figure 11. The addition of 3 wt.% NS caused a significant decrease in crack length and total cracking area by 25% in comparison with the reference samples [22]. Even small amounts of NS can improve the mechanical performance of concrete in a similar way as that of high volumes of SF. It is important to note that NS has a negligible effect on shrinkage development, whereas SF increases it in early age [111].

**Figure 12:**
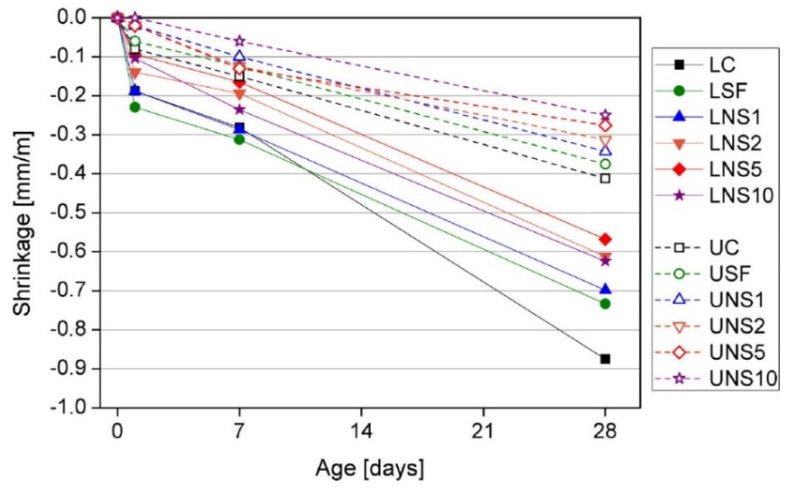
Experimental results of a drying shrinkage test of lightweight concrete (LC) and ultra-lightweight concrete (UC), and concretes with cement replacement by 1, 2, 5, ad 10 wt.% of NS and 10 wt.% of silica fume (LSF and USF), reprinted with permission from ref. [111].

Research presented by Sikora et al. [111] showed that with increasing the addition of fine materials (silica fume or nanosilica), the final drying shrinkage (after 28 days) decreases. The strongest effect, as shown in Figure 12, can be observed for 1–5 wt.% NS—later this influence diminishes. The positive effect of NS addition can be attributed to the improvement of ITZ and the bonding strength between the aggregate and cement paste, which hinders water movement.

## 4. Influence of Nanosilica on Microstructure and Durability-Related Properties

Nanosilica was found to have a substantial effect on the refinement of the pore structure of cement paste; thus, produced NWC is less porous and permeable [104,112]. However, this phenomenon is still discussed in the case of LWAC, as the porous nature of the aggregate is believed to be responsible for the lower durability compared with ordinary concrete. The porous structure of LWA causes the concrete to be more prone to ingress of harmful substances, which leads to a generally lower durability. The use of LWA also leads to a change in the pore structure and porosity of concrete in comparison with normal-weight concrete. This is why not all of the results and effects of nanosilica in NWC can be directly applied for LWC. Because of this, separate studies have been carried out, of which the most recent and worth mentioning are summarized in Table 3.

**Figure 13:**
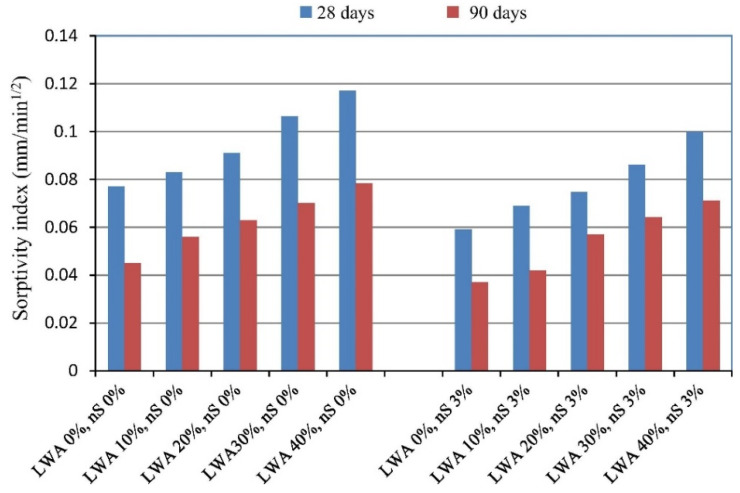
The effect of LWA and NS content on the sorptivity index of high-strength LWC. Reprinted with permission from ref. [96] Copyright 2017, Elsevier.

When analysing above-mentioned articles, a common conclusion can be drawn that the NS particles reduce the size of capillary pores and modify the pore structure. According to Atmaca N. et al. [96], the reduced sorptivity values prevent the penetration of aggressive solutions to the pore structure (Figure 13). This is caused by the creation of finer pores after adding NS. Nanoparticles also increase the hydration process in cement paste because of the pozzolanic reaction, which leads to a lower number of continuous capillary pores, which correspond to a lower water absorption [112]. This effect was confirmed by Vargas P et al. [7]. The researchers indicated that nanosilica can reduce the pore volume in LWC by up to 3% in aliven concrete and 3.3% in perlite. In both concretes, the addition of 10 wt.% NS led to a decrease in water absorption. It is safe to say that both the pore volume and the water absorption of the LWC could be reduced with the addition of NS, but the final effect depends mainly on the type of LWA used.

**Figure 14:**
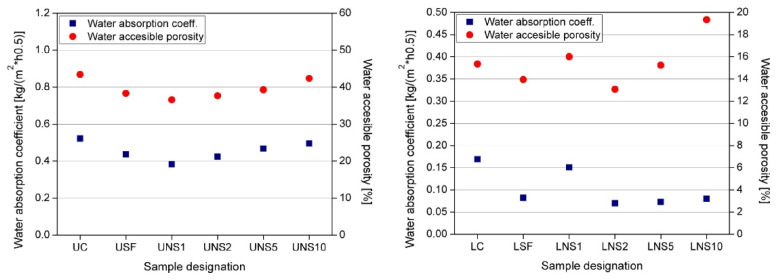
Water accessible porosity and water absorption coefficient of ultra-lightweight (**left**) and lightweight concretes (**right**) modified with 10 wt.% of silica fume (SF) and 1, 3, 5, and 10 wt.% NS after 28 days of curing, reprinted with permission from ref. [111].

Another influence of NS on the microstructure of cement composites was reported by Wang X.F. et al. [22]. They found that the ITZs of LWAC with 3 wt.% nano-SiO_2_ were more compact than that of the reference LWAC. Similar results were obtained by Elrahman M.A. et al. [72]. For a composite modified with nano-SiO_2_, the border between the paste matrix and the ITZ could not be measured. This, on the other hand, could lead to a water penetration depth that decreased by up to 43% according to Du H. [47]. The addition of NS reduced the open porosity thanks to the filling effect and an increase in the produced hydrates from the pozzolanic reaction between NS and Ca(OH)_2_ [19]. These phenomena were analysed in [111], resulting in the data presented in Figure 14.

**Figure 15:**
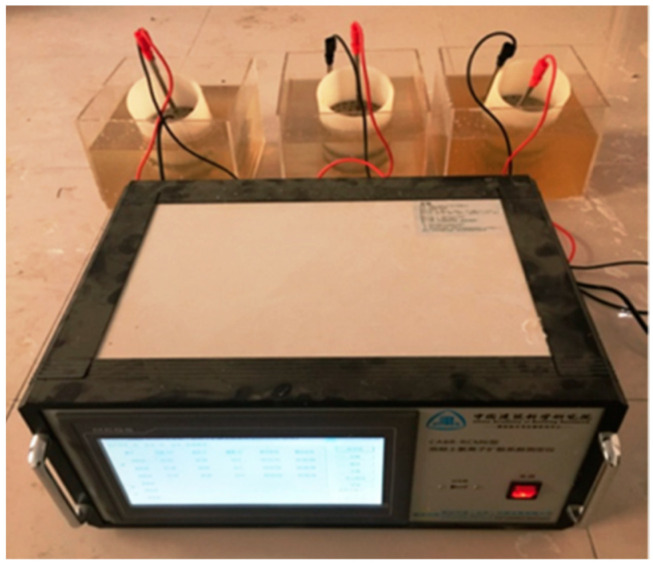
Test device for the rapid chloride migration (RCM) method, reprinted with permission from ref. [117].

**Figure 16:**
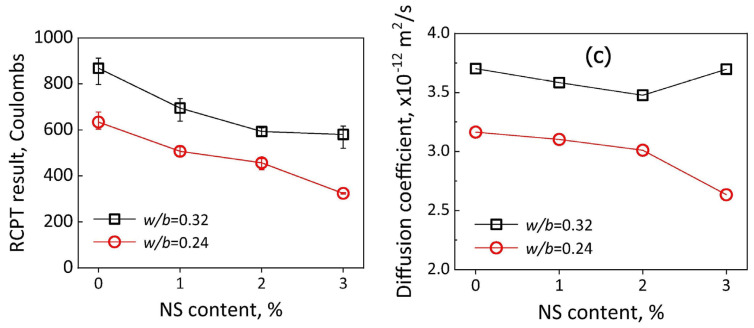
RCPT results (**left**) and chloride diffusion coefficients (**right**) of lightweight concrete modified with 1, 2, and 3 wt.% NS. Reproduced with permission from ref. [19] Copyright 2019, Elsevier.

Another often applied method that allows for evaluating the durability of concrete is the ultrasound pulse velocity. Higher velocities obtained from the test indicate that the tested specimen has a low porosity, and thus a potentially higher durability [113]. For instance, Sadeghi Nik et al. [114] reported that adding 2 wt.% and 4 wt.% nanosilica resulted in an increased ultrasonic pulse velocity, while higher amounts (6 wt.%) would not cause any significant increase. Another important parameter measured to determine the durability is the electrical resistivity of concrete, which reflects how concrete limits the movement of ions [23,115]. Studies done by Hornbostel et al. [116] showed that the rate of corrosion increases with the decrease in electrical resistivity. The results of the electrical resistivity tests show that use of NS more than doubles the electrical resistivity, consequently decreasing the probability of corrosion occurrence. Other tests that can help to determine the durability of an element are the Rapid Chloride Penetration Test (RCPT) and Rapid Chloride Migration (RCM). Typical measurement equipment for RCM is shown in Figure 15. In principle, the results of these tests represent the electrical conductivity (or resistivity) of concrete, and the results obtained by Du are presented in Figure 16 [19]. For OPC concrete, there is a clear relationship between the concrete’s resistance against chloride and its electrical conductivity.

The study of Du et al. [95] on the effect of the NS dosage (up to 3 wt.%) confirmed a beneficial effect of NS on LWC resistance against chloride ion penetration. RCPT confirmed (Figure 16) that the inclusion of NS resulted in a substantial reduction of electrical conductivity, as well as a diffusion coefficient for the different rates of water-to-cement ratios.

**Figure 17:**
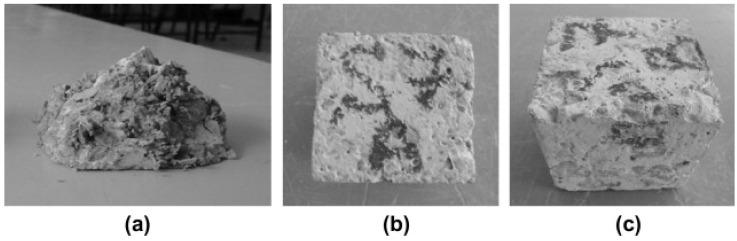
After 300 cycles of freeze and thaw: (**a**) control sample, (**b**) sample containing 5 wt.% nanosilica, and (**c**) sample containing 3 wt.% nanoalumina, reprinted from [118] with permission from Elsevier, 2013.

As reported by researchers, NS reduces the size of capillary pores, changes the pore structure, and reduces overall sorptivity [19,22,72,95]. This results in increased durability of the produced elements, which can be indirectly measured by checking the pulse velocity or electrical resistivity of the hardened concrete. Unfortunately, only a limited number of studies focus on the freeze−thaw resistance and corrosion resistance of NS-modified LWCs. A study on the freeze−thaw resistance of normal-weight concrete was already conducted by Behfarnia et al. [118]. The results presented in Figure 17 that show the control sample (Figure 17a) and samples with nanomaterials (Figure 17b,c) after 300 freeze−thaw cycles clearly indicate the influence of the added nanomaterials.

**Figure 18:**
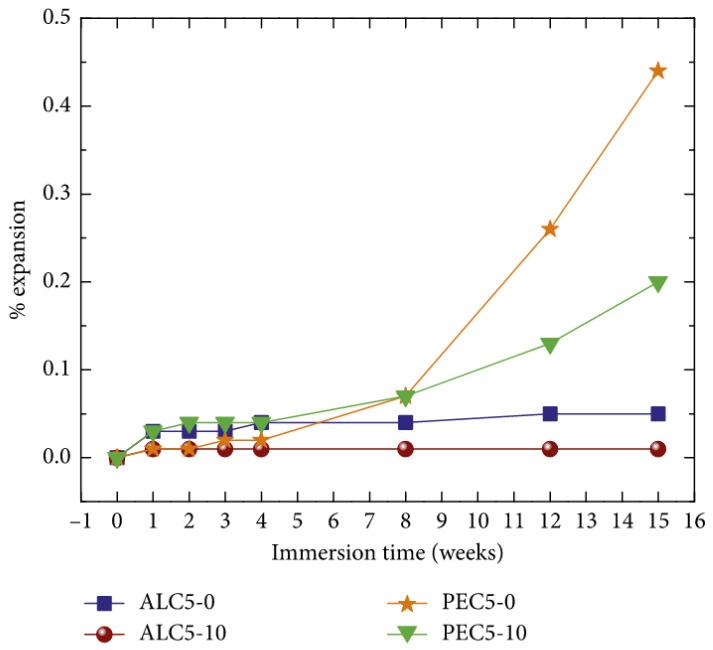
Expansion of LWCs with and without NS produced with two types of lightweight aggregates under sulfate attack. Reprinted with permission from ref. [7].

Vargas et al. [7] evaluated the expansion of lightweight concretes produced with two types of LWAs (aliven and perlite) with (10 wt.%) and without NS (Figure 18) under sulfate attack (MgSO_4_). For up to 4 weeks, all of the specimens exhibited comparable expansion (Figure 18). Afterwards, clear differences were observed for different LWAs and NS amounts. The specimens containing the perlite aggregate (PEC5-0, PEC5-10) exhibited significantly higher expansion values than the LWC produced with the aliven aggregate (ALC5-0 and ALC5-10). The LWC produced with the perlite aggregate presented an increasing expansion for longer immersion times in sulfates, while in the case of the LWC containing aliven, no further increment of expansion was reported. The LWCs with the addition of 10% of nanosilica (PEC5-10 and ALC5-10) exhibited a lower expansion under the attack of magnesium sulfate. In the 15 weeks of testing, expansion of the reference PEC5-0 reached 0.44%, while NS-modified LWC (PEC5-10) exhibited an expansion of 0.2%. This effect was attributed to the previously described refinement of the concrete pore structure due to the NS presence, which resulted in decreased water absorption and voids volume. Moreover, because of the reaction of CH with NS, a lower CH content in the NS-modified LWCs was present. This, in turn, resulted in the limited possibility of brucite formation during the sulfate attack.

## 5. Conclusions

The use of nanosilica in LWC refines the pore structure of the cementitious matrix and the ITZ by increasing the formation of C-S-H. This densification of the cementitious matrix leads to a smaller porosity and lower water absorption. Depending on the study, the use of NS led to increases in the compressive strength of lightweight concrete of up to 20%. It is also proven that nanosilica is more efficient than silica fume, even in smaller dosages. It is recommended to limit the amount of nanosilica to maximum of 5 wt.% cement in order to obtain the optimal improvement for all of the properties. On the other hand, it has to be remembered that adding nanosilica to a concrete mixture always decreases the workability and flowability. To maintain workability and flowability, a significant amount of PCE superplasticizer needs to be used, often exceeding manufacturer recommendations about dosage. Even though NS is not a new material and has been studied in recent years, there are still areas that need to be properly investigated, especially regarding its use in lightweight concrete. There were only a few papers where the freeze−thaw resistance or influence of different artificial aggregates were investigated. Intensive investigation needs to be done on all aspects of durability in the context of possible 3D printing with lightweight concrete modified with nanosilica particles.

Academic studies should result not only in a theoretical knowledge, but should provide practical and industrial applications. This is why LWCs with NS need to be further investigated, as current knowledge indicates that nanomaterials can be successfully incorporated in concrete prefabrication technology. Based on recent papers, it is already known that LWCs have a different structure, pore system, and mechanical and physical properties, but with addition of NS, all of their disadvantages can be minimalized. What needs to be investigated in the foreseeable future is the scale effect, cost analyses in terms of industrial application, and modification of LWCs with NS to make them suitable for 3D printing. Until now, most of studies have been conducted on standard laboratory samples, which does not reflect the behaviour of mass elements. In many studies the amounts of PCE plasticizers in mixes were significantly increased, which limits the use of these mixes outside the laboratory because of elevated costs. This is why LWCs with NS need to be investigated on a bigger scale with economic evaluation.

## Figures and Tables

**Table 1:** Influence of nanosilica on rheological properties.

Research	Analysed Properties	Results
Du et al. [95]	Slump flow test	To maintain slump flow, the superplasticizer needed to be increased by 100% for each 1 wt.% of NS
Atmaca et al. [96]	Slump flow test	To maintain slump flow, the superplasticizer needed to be increased by 33% for 3 wt.% of NS
Naniz et al. [23]	Slump flow test	Slump flow decreased by about 13%-17% with 5 wt.% NS dosage, depending on w/c
	J-Ring test	Flow diameter decreased by 15% for 5 wt.% NS, regardless of the w/c ratio
	U-box test	Height difference increased by 150–500% for 5 wt.% NS, but still met the criteria for flowability
	V-funnel flow test	Flow time increased by 31–98% for 5 wt.% NS, even though the SP dosage increased
Du [19]	Flow test	To maintain flowability, 40–80% SP was added for 3 wt.% NS based on the w/c ratio
Yu et al. [27]	Slump and flow test	To maintain consistency with 10 wt.%, the NS dosage required an additional 1% SP, counted as the wt.% of the binder

**Table 2:** Influence of NS on the mechanical properties.

Research	Analysed Properties	Results
Du et al. [95]	Compressive strength	Best effect for 2 wt.% NS on early strength (7 days), up to a 13% increment
Wang et al. [22]	Compressive strength	For 3 wt.%, NS compressive strength increased by 23.5%, 23.7%, and 16.8% for 3, 7, and 28 days, respectively
Atmaca et al. [96]	Compressive strength Splitting tensile strength	For 3 wt.%, NS compressive strength increased by 12.9% and 10.7% for compressive and splitting tensile strength, respectively, after 90 days
Naniz et al. [23]	Compressive strength	For 3 wt.%, NS increased by 19.2–21.0%, 18.0–18.8%, and 20.0–24.0% for compressive, splitting tensile, and flexural strength, respectively, depending on the w/c ratio
	Splitting tensile strength	
	Flexural strength	
Du [19]	Compressive strength	For 2 wt.%, NS compressive strength increased by 31% after 28 days
Yu et al. [27]	Compressive strength	For 10 wt.%, the NS compressive strength increased by 21%
Vargas et al. [7]	Compressive strength	For 10 wt.%, the NS compressive strength decreased by 16%
Zhang et al. [73]	Compressive strength Flexural strength	For 0.1 wt.%, the NS increased by 40% and 18% compressive and flexural strength, respectively
Abd Elrahman et al. [72]	Compressive strength Flexural strength	For 4 wt.%, the NS increased by 16% and 25% compressive strength, and 26% and 31% flexural strength after 7 and 28 days, respectively

**Table 3:** Influence of NS on durability-related properties.

Research	Analysed Properties	Results
Du et al. [95]	Accessible water porosity	Best effect for 1 wt.% NS, 15% to 17% reduction based on the cement type
	Water sorptivity	Best effect for 1 wt.% NS, 35% reduction for OPC
	Water penetration depth	Optimal dosage of 1 wt.%, slag cement helped to reduce the depth penetration
	Rapid chloride penetration and Rapid chloride migration	The more NS, the stronger effect, optimal result for 2 wt.% with chloride migration coefficient reduced by 36%
	Chloride diffusion	2 wt.% NS reduced the diffusion coefficient up to 20%
Wang et al. [22]	Microstructure with SEM	ITZ’s microstructure for 3 wt.% NS was more compact than the reference
Atmaca et al. [96]	Water sorptivity	The sorptivity coefficient decreased by 17–23% with 3 wt.% NS based on the cement type
	Gas permeability	Reduction up to 30% after 28 days and up to 40 after 90 days for 3 wt.% NS
Naniz et al. [23]	Ultrasonic pulse velocity	Velocity decreased by 2.0–2.7% regarding the w/c ratio for 3 wt.% NS
	Electrical resistivity	Electrical resistivity increased by 195% and 304% regarding w/c for 5 wt.% of NS, equivalent to changing the corrosion rate from very high to low to moderate
Du [19]	Water accessible porosity	2 wt.% and 3 wt.% NS addition reduced the porosity by 3.3% and 2.8%, respectively
	Water penetration depth	2 wt.% NS reduced the depth penetration by 43%
	Water sorptivity	The sorptivity coefficient decreased by 30% with 3 wt.% of NS
	Rapid chloride penetration	The more NS, the stronger effect, best result for 3 wt.% with chloride migration coefficient reduced by 49%
	Chloride diffusion	Best result for 2 wt.% NS, regardless of the w/c ratio
Yu et al. [27]	Thermal conductivity	Negligible effect of NS on thermal conductivity
Vargas et al. [7]	Pore volume and water absorption	For 10 wt.%, the NS pore volume decreased by 3% and absorption decreased by 4.5%
Abd Elrahman et al. [72]	Thermal conductivity	No significant effect for 0–4 wt.% NS
	Effective water porosity and water absorption	4 wt.% dosage of NS decreased WAC over four times and decreased the effective water porosity from 19% to 7%
	Air-void characteristics	With 2 wt.% NS, the number of voids was significantly lower
	Mercury intrusion porosimetry	With 4 wt.%, the NS total porosity went from 54% to 39%

## Data Availability

The data presented in this study are available upon reasonable request from the corresponding author.
